# Toxigenicity of *F. graminearum* Residing on Host Plants Alternative to Wheat as Influenced by Environmental Conditions

**DOI:** 10.3390/toxins14080541

**Published:** 2022-08-08

**Authors:** Sigita Janaviciene, Skaidre Suproniene, Grazina Kadziene, Romans Pavlenko, Zane Berzina, Vadims Bartkevics

**Affiliations:** 1 Institute of Agriculture, Lithuanian Research Centre for Agriculture and Forestry, LT-58344 Akademija, Lithuania; 2 Institute of Food Safety, Animal Health and Environment “BIOR”, Lejupes Iela 3, LV-1076 Riga, Latvia

**Keywords:** alternative host, *Fusarium graminearum*, mycotoxin profile, environmental conditions

## Abstract

*Fusarium graminearum* is an important pathogen that causes Fusarium head blight (FHB) in several cereal crops worldwide. The potential of this pathogen to contaminate cereals with trichothecene mycotoxins presents a health risk for both humans and animals. This study aimed to evaluate the potential of different trichothecene genotypes of *F. graminearum* isolated from an alternative host plant to produce mycotoxins under different spring wheat grain incubation conditions. Fourteen *F. graminearum* strains were isolated from seven alternative host plants and identified as 3-acetyl-deoxynivalenol (3-ADON) and 15-acetyl-deoxynivalenol (15-ADON) genotypes. These strains were cultivated on spring wheat grains at 25 °C and 29 °C for 5 weeks. The mycotoxins produced were analysed with a high-performance liquid chromatograph (HPLC) coupled to a Thermo Scientific TSQ Quantiva MS/MS detector. The obtained results showed that the *F. graminearum* strains from alternative host plants could produce nivalenol (NIV), deoxynivalenol (DON), fusarenon-X (FUS-X), 3-ADON, deoxynivalenol-3-ß-d-glucoside (D3G), 15-ADON, and zearalenone (ZEA). *F. graminearum* strains produced DON and ZEA under both temperatures, with the mean concentrations varying from 363 to 112,379 µg kg^−1^ and from 1452 to 44,816 µg kg^−1^, respectively. Our results indicated the possible role of dicotyledonous plants, including weeds, as a reservoir of inoculum sources of *F. graminearum*-induced Fusarium head blight, associated with the risk of mycotoxin contamination in spring wheat.

## 1. Introduction

Wheat is the third most important crop in the world, with annual production in 2021 amounting to 770 million tons [1], which should increase in the future to meet the growing demand resulting from the rising human population. This aligns with the United Nations Sustainable Development Goal of promoting sustainable agriculture that provides sufficient, safe, and high-quality food by 2030 [2].

Climate change poses serious challenges to global food security. Long-term changes in the temperature, humidity, precipitation, and extreme weather influence farming practices and the quality of food crops. The susceptibility of microorganisms, especially those producing toxins and other pests, to climate factors suggests that climate change may promote the occurrence and severity of some foodborne diseases [3]. Food security depends on the resistance of the main food crops to climate change and extreme conditions, but the resistance of wheat to these factors in Europe remains unknown [4]. In wheat, a 1 °C rise in minimum or maximum temperatures during the cropping season could decrease global wheat production by ~5.6% [5]. Crop losses of many types of cereals due to climate change and fungal infections are among the most important concerns worldwide. *Fusarium graminearum* is primarily perceived as an agricultural pathogen affecting monocotyledonous plants, but its hosts also include dicotyledonous plants and various weeds in the agricultural environment. In the absence of host plants, weeds may serve as reservoirs of high genetic diversity and provide sources of Fusarium head blight (FHB) infection for host plants [6,7,8]. Exposure to climate change can dramatically increase FHB infection and thus the concentration of deoxynivalenol (DON) mycotoxin in wheat and processed food products, which may pose a health risk to humans and animals [8]. 

Mycotoxins are widely distributed throughout the world [3,8,9,10,11]. It has been reported that 25–50% of the world’s crop yield is contaminated with mycotoxins yearly [10,12,13]. Mycotoxins are frequently found in Lithuania-grown grains of spring cereals [14,15,16]. One of the most prevalent mycotoxins in Lithuania and other European countries is type B trichothecene DON. However, factors promoting the formation of its metabolites 3-acetyl-deoxynivalenol (3-ADON) and 15-acetyl-deoxynivalenol (15-ADON) have not been sufficiently elucidated. Most studies on the genotypes and chemotypes of *F. graminearum* populations were carried out using fungal strains isolated from the primary host plants wheat, barley, and other small-grained cereals [11,17,18]. A comprehensive analysis of trichothecene genotypes and population diversity of *F. graminearum* strains isolated from alternative host plants (oilseed rape, sugar beet, and various weeds) was conducted in Lithuania in 2015–2018 [6]. This study demonstrated the ability of *F. graminearum* isolated from all asymptomatic dicotyledonous hosts to induce FHB in spring wheat [7]. The distribution of DON and its metabolites in spring cereals has been investigated to date. Still, the information on the ability of *F. graminearum* from asymptomatic host plants to produce trichothecenes is not yet available. Therefore, it is especially important to clarify the situation in this regard, as the toxicity of the metabolites of DON is different [19,20]. The dominant mycotoxins produced by *F. graminearum* are zearalenone (ZEA), DON and their derivatives; these mycotoxins are often identified in different cereals contributing to the reduction in grain quality and safety [21,22,23,24]. The presence of mycotoxins lowers grain prices and poses serious risks to human and animal health [25,26]. Scientists worldwide working on food quality and safety are seeking to develop a flexible modeling approach to mycotoxin risk assessment. This includes the effects of climate change (temperature, humidity, etc.) on the production of mycotoxins and their occurrence in food [20]. 

The potential for trichothecene production is still unclear, depending on how different environmental conditions may influence mycotoxin production and what the effects of environmental stress are on the occurrence profiles of secondary metabolites. It is widely known that different mycotoxin-producing fungal species differ in their preferred climatic conditions, leading to a wide variation in mycotoxin distribution worldwide. Climate change is expected to influence the spread of microscopic fungi, lead to changes in agricultural practices, and affect the distribution and levels of mycotoxins in crops [27]. A better understanding of the role of individual factors in the production of mycotoxins would help to assess the risks to food safety and to design preventive strategies in anticipation of climate change.

This research aims to evaluate the potential of different genotypes of *F. graminearum* isolated from alternative host plants to produce mycotoxins upon incubation on spring wheat grains under different conditions. 

## 2. Results

**Figure 1:**
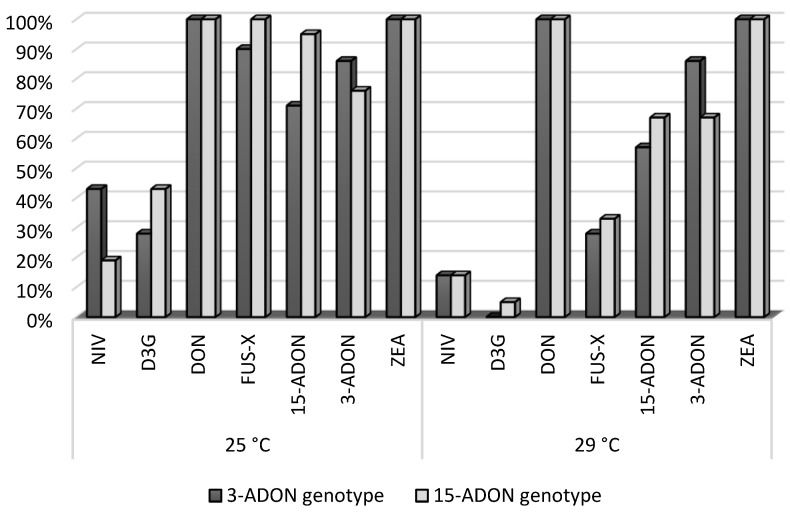
The percentage of spring wheat grain samples contaminated with mycotoxins depending on the temperature and trichothecene genotype.

All strains of *F. graminearum* were found to grow mycelium on spring wheat grains and to produce particularly high levels of mycotoxins in the grains. The spring wheat grains were inoculated with *F. graminearum* strains that produced NIV, DON, FUS-X, 3-ADON, D3G, 15-ADON, and ZEA. All the spring wheat grain samples (100%) were contaminated with DON and ZEA (Figure 1). 

The investigated *F. graminearum* strains produced DON and ZEA under both conditions tested, with the mean concentrations varying from 363 to 112,379 µg kg^−1^ and from 1452 to 44,816 µg kg^−1^, respectively (Table 1). 15-ADON was detected in between 57% and 95% of the samples, and 3-ADON was detected in between 67% and 86% of the samples, depending on the incubation conditions and trichothecene genotype. 15-ADON and 3-ADON were produced in lower amounts, and the mean concentrations varied from <50 to 22,246 µg kg^−1^ and from <50 to 28,800 µg kg^−1^, respectively. There was some distinction between ZEN, DON, 3-ADON, and 15-ADON production among the *F. graminearum* strains, i.e., the strain of *Triticum aestivum* (6K4V1) was the most potent producer of ZEN, whereas the *Poa annua* L (1350s) and *Tripleurospermum inodorum* (L.) Sch. (1120p) strains were mainly responsible for the increased 3-ADON and 15-ADON production, respectively. All strains were the most effective producers of DON, with the highest amount of DON produced by the *Brassica napus* L. (425l) strain at the concentration of 223,532 µg kg^−^^1^. In the spring wheat grain samples, NIV was detected between 14% and 43%, while D3G in between 0% and 43% of samples, depending on the incubation temperature and trichothecene genotype. NIV and D3G were detected at very low concentrations or not detected at all. The highest NIV and D3G concentrations were 3015 µg kg^−1^ and 187 µg kg^−^^1^, respectively. Importantly, FUS-X was detected in between 28% and 100% of samples, with concentrations varying from <10 to 220 µg kg^−1^. 

The simultaneous presence of DON and ZEA was found in 100% of the wheat samples incubated at both temperatures using both trichothecene genotype strains. Higher concentrations were found in grain samples incubated at 25 °C and inoculated with 15-ADON trichothecene genotype strains. However, the strains of the 3-ADON genotype also produced relatively high concentrations of DON and ZEA. It should be noted that elevated concentrations of DON and ZEA were also produced at 29 °C.

The strains of the 3-ADON genotype incubated at 25 °C temperature produced NIV, FUS-X, and 3-ADON at higher concentrations than the strains of the 15-ADON genotype (Table 1). The strains of the 15-ADON genotype produced DON, ZEA, 15-ADON, and D3G at higher concentrations than the strains of the 3-ADON genotype. The strains of the 3-ADON genotype incubated at 29 °C produced NIV and 3-ADON at higher concentrations than those of the 15-ADON genotype. The strains of the 15-ADON genotype produced DON, ZEA, 15-ADON, FUS-X, and D3G at higher concentrations than the 3-ADON genotype strains.

**Figure 2:**
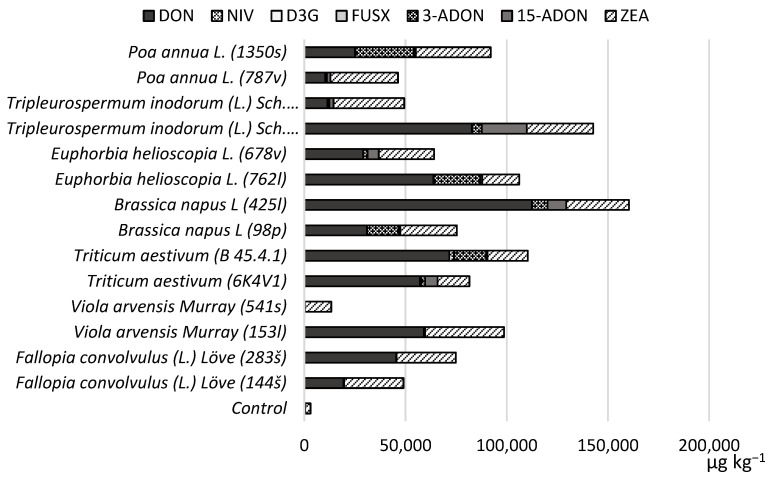
The average produced mycotoxin concentrations (µg kg^−1^) in spring wheat grains with alternative host strains incubated at 25 °C.

Regarding the strain-dependent potential for mycotoxin production, we found that all strains can produce DON in spring wheat grains incubated at 25 °C. Figure 2 shows the mycotoxin production potential and concentrations compared to the control sample. All strains are potential producers of DON and ZEA in spring wheat grain. *Poa annua* L. (1350s) and *Euphorbia helioscopia* L. (762l) strains produced higher concentrations of 3-ADON than the strain isolated from the primary host plant—*Triticum aestivum* (B 45.4.1). Some of the highest concentrations of 15-ADON were produced by the *Tripleurospermum inodorum* (L.) Sch. (1120p) strain. The presence of NIV was also detected, but the average concentrations were lower, ranging from <100 to 2273 µg kg^−1^. Overall, the strain of *Brassica napus* L (425l) produced the highest amounts of toxins. 

**Figure 3:**
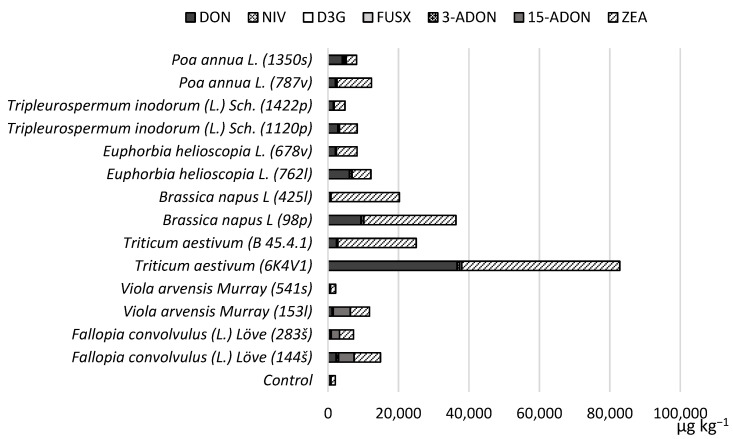
The average produced mycotoxin concentrations (µg kg^−1^) in spring wheat grains inoculated with alternative host strains and incubated at 29 °C.

Slightly different results were obtained after the spring wheat grains were incubated at 29 °C. This temperature was less favorable for producing particularly high concentrations of mycotoxins in spring wheat grains inoculated with *F. graminearum* strains obtained from alternative host plants. However, the *Triticum aestivum* (6K4V1) strain produced higher DON and ZEA levels at this temperature (Figure 3). It should be noted that under these conditions, this strain produced higher amounts of ZEA in grains compared to those incubated at 25 °C. Additionally, both *Brassica napus* L strains (425l, 98p) produced higher concentrations of ZEA*. Fallopia convolvulus* (L.) Löve (144š, 283š) and *Viola arvensis* Murray (153l) strains produced higher levels of 15-ADON and FUS-X at 29 °C compared to the samples incubated at 25 °C. 

The results of the analysis of variance are summarized in Table 2. The statistical analysis (ANOVA) showed that the identity of *F. graminearum* strains (Factor A) had a significant effect on the production of DON (*p* < 0.01), 3-ADON (*p* < 0.0001), 15-ADON (*p* < 0.001), and ZEA (*p* < 0.0001). The incubation temperature (Factor B) was significant for the production of DON (*p* < 0.0001), 3-ADON (*p* < 0.0001), 15-ADON (*p* < 0.001), D3G (*p* < 0.01), and ZEA (*p* < 0.0001). The combined effects of all the independent variables contributed significantly to the production of 3-ADON and 15-ADON (*p* < 0.001).

The statistical analysis showed that the strains of *F. graminearum* and the incubation temperature were insignificant in the production of NIV and FUS-X.

In our study, significant positive correlations were found between DON and 15-ADON, NIV and D3G, FUS-X, D3G and FUS-X, and between 3-ADON and 15-ADON in spring wheat grains incubated at 25 °C and inoculated with 15-ADON genotype strains. (Table 3 (left)). Apart from the relationships between some of the mycotoxins, the strongest positive correlation was observed between NIV and FUS-X (r = 0.991). Significant negative correlations were observed between NIV, D3G, FUS-X, and ZEA, with the strongest negative correlation between FUS-X and ZEA (r = −0.882). 

Correlations between individual mycotoxins in spring wheat grains incubated at 25 °C and inoculated with the 3-ADON genotype were observed in the cases of NIV and DON, D3G and DON, NIV, as well as FUS-X and NIV, D3G, ZEA and 15-ADON. The strongest positive correlation was observed between D3G and NIV (r = 0.970) (Table 3 (right)).

Significant positive correlations between DON, D3G and 3-ADON, ZEA, FUS-X and 15-ADON, 3-ADON and ZEA were found in spring wheat grains incubated at 29 °C and inoculated with the 15-ADON genotype strains (Table 4 (left)). The strongest positive correlation was observed between DON and 3-ADON (r = 0.984).

Correlations between individual mycotoxins in spring wheat inoculated with the 3-ADON genotype and incubated at 29 °C were observed in the cases of DON and 3-ADON and between ZEA, DON and 3-ADON. The strongest positive correlation was observed between DON and 3-ADON (r = 0.834) (Table 4 (right)).

## 3. Discussion

In this study, we evaluated the potential of different trichothecene genotypes of *F. graminearum* isolated from alternative host plants to produce mycotoxins under different conditions of spring wheat grain incubation. In most parts of the world, *F. graminearum* is the predominant FHB-causing species [28]. Earlier studies have shown that *F. graminearum* isolates could be identified as 3-ADON and 15-ADON trichothecene genotypes. Out of the 210 *F. graminearum* isolates obtained from Lithuanian weeds and assessed for trichothecene genotype, 154 isolates (73.3%) were identified as the 15-ADON genotype, and 49 isolates (23.3%) belonged to the 3-ADON genotype. None of the isolates in that study corresponded to the NIV genotype [7]. Trichothecene genotype structure in alternative host plants appeared to be very similar to that of spring wheat (73%—15-ADON, 26%—3-ADON, and 1%—NIV) [29] and reflected the overall distribution of *F. graminearum* chemotypes from cereals in Europe [30,31,32]. Therefore, the 15-ADON and 3-ADON trichothecene genotypes were also selected for our study. The 15-ADON genotype is prevalent in most European countries, while the 3-ADON genotype has achieved greater prevalence in parts of North America [33]. The aggressiveness of the 3-ADON and 15-ADON genotypes varies in wheat. Some studies have demonstrated that the 3-ADON genotype is more aggressive and produces higher amounts of trichothecenes than the 15-ADON genotype [18,34]. Still, other studies reported no difference in aggressiveness between wheat’s 3-ADON and 15-ADON genotypes [35,36]. In the present study, *F. graminearum* 15-ADON genotype strains were more aggressive and produced more mycotoxins in wheat grain than *F. graminearum* 3-ADON genotype. It has been reported that trichothecenes are important factors in the aggressiveness of FHB disease in wheat and other cereal crops [37]. Isolates of the 3-ADON genotype have been shown to produce higher levels of FHB and trichothecenes in wheat, grow faster, and produce conidia more abundantly on the nutrient media than isolates with the 15-ADON genotype [18,38]. Our study observed that the levels of mycotoxin production depended not only on the trichothecene genotype but mostly on the strain and environmental conditions. It should be noted that isolates producing higher levels of mycotoxins and having the 15-ADON genotype were also found in our study. Generally, *Fusarium graminearum* isolates vary in their ability to cause disease in different hosts [39].

Based on their trichothecene production, *F. graminearum* strains can be divided into three genotype groups: the 15-ADON genotype produces DON and 15-ADON, the 3-ADON genotype produces DON and 3-ADON, and the NIV genotype produces NIV and 4ANIV [40]. Seojin Ahn et al. [41] showed that few *Fusarium* isolates produced NIV, 3-ADON, and 15-ADON mycotoxins. In our study, some of the isolates produced NIV, 3-ADON, and 15-ADON, but also DON and ZEA in combination. Different isolates produced different combinations of mycotoxins. The mycotoxin profile was affected not only by the identity of strains but also by the environmental conditions. Lithuania has a humid continental climate (Dfb in the Köppen climate classification) [42]. Climate change is expected to seriously affect wheat (*Triticum aestivum* L.) production around the world in the future [43]. Wheat grain yield is predicted to decrease because of the global increase in air temperature [44]. The Fusarium chemotypes can vary annually depending on weather conditions. The weather conditions of the alternative host plants’ collection times differed in 2015 and 2016. The summer period, especially August, was very dry and warm in 2015 compared to the summer of 2016, which was windy and warm. The beginning of summer was dry, later wet. Comparing the meteorological conditions in Lithuania each year with the long-term average of 1924–2018, the climate is warming, and the average temperature has increased by 1.4 °C. Precipitation remained similar on average. Climate warming is a very important factor in the spread of plant diseases and the emergence of mycotoxins. Fungal spores can be transported from the soil surface to the head via rain splash dispersal [45,46,47].

Deoxynivalenol and ZEN are two of the most relevant mycotoxins for the agri-food industry and the human food supply. Additionally, in our study, these mycotoxins were produced by all isolates at both temperatures. However, the mycotoxin accumulation potential may be a population-specific feature, not tied directly to the trichothecene genotype. Thus, we demonstrated that the Lithuanian *F. graminearum* alternative host plants with trichothecene 3-ADON and 15-ADON genotypes could produce not only large amounts of trichothecenes but also significant levels of ZEA. Previous studies by Mylona et al. [48] showed that at 30 °C, there was a significant reduction in the production of ZEN in stored wheat grain, indicating that intermediate temperatures of 15–25 °C may be more relevant to ZEN contamination of cereal-based commodities. In our study, the average amount of ZEA was higher at 25 °C, but several strains produced much more ZEA at 29 °C.

The potential of *F. graminearum* to produce DON, 15-ADON, 3-ADON, NIV, D3G, FUS-X, and ZEA was determined in our study. To our knowledge, this is the first report regarding mycotoxin production on spring wheat grains by *F. graminearum* strains isolated from alternative host plants in Europe.

Depending on environmental factors, *F. graminearum* can survive on crop residues, grow, and produce conidia and sexual structures, which provide the primary inoculum causing disease on wheat heads and later the production of secondary metabolites known as mycotoxins [49]. In the present study, *F. graminearum* isolates were able to cause FHB disease in spring wheat regardless of their chemotypes. This result strongly suggests that dicotyledonous plants, including weeds, can serve as alternative hosts for FHB-causing species, particularly *F. graminearum*, in spring wheat.

## 4. Conclusions

Strains with 3-ADON genotype consistently produced higher concentrations of 3-ADON, and the strain with 15-ADON genotype produced more 15-ADON in spring wheat grains after 5 weeks of incubation. However, the production of DON, ZEA, NIV, 15-ADON, and 3-ADON in spring wheat grains was more often dependent on the strain of *F. graminearum* and the environmental conditions than the trichothecene genotype.

Our results point to the potential role of weeds and dicotyledonous plants as a reservoir of inoculum sources of *F. graminearum*-induced FHB and the risk of mycotoxin contamination in spring wheat grain.

In the context of global warming trends, it is very important to note that the optimal conditions for ZEA and DON occurrence in crops have a wide range, and these mycotoxins can be formed under warmer conditions.

Appropriate weed control in the croplands is necessary as they could serve as potential hosts for *Fusarium graminearum,* which poses a risk of spring wheat fusarium head blight and grain contamination with mycotoxins.

## 5. Materials and Methods

### 5.1. Sample Collection 

Alternative host plants were collected from fields located in Central Lithuania (55°23′50″ N, 23°51′40″ E) from 2015 to 2016 and had an endocalcic-epihypogleyic cambisol soil type. Asymptomatic weed plants were collected in August and September 2015 from non-cereal crops and in July 2016 from cereal crops. The trichothecene genotype of *F. graminearum* strains was identified in previous studies [7]. The ability of *F. graminearum* strains obtained from different host plants to produce mycotoxins in spring wheat grain was tested in experiments conducted in 2021 at the Institute of Agriculture, Lithuanian Research Centre for Agriculture and Forestry (Akademija, Kėdainiai distr., Lithuania). 

A total of 14 *F. graminearum* strains comprising 10 *F. graminearum* strains were obtained from asymptomatic weeds, 2 from the primary host plant spring wheat (6K4V1; B 45.4.1), and 2 from spring oilseed rape (98p; 425l), were tested for their ability to produce mycotoxins in spring wheat grain. A total of 15 treatments were tested in triplicate. The control samples were not inoculated. The study scheme is presented in Table 5. 

### 5.2. Revitalization of F. graminearum Isolates

Cryotubes containing spore suspensions of Fusarium isolates selected for the testing were removed from the freezer (−80 °C) and stored in a refrigerator for one day to allow the spore suspension to thaw slowly. A 10 µL aliquot of the spore suspension was spread using an L-shaped sterile Drigalski spatula on a water agar medium and incubated for 2 to 3 days at 22 ± 2 °C. When individual colonies appeared, they were transferred onto potato dextrose agar (PDA) medium, and the plates were incubated at 22 ± 2 °C for 7 days.

### 5.3. Spring Wheat Grain Inoculation under Laboratory Conditions

The spring wheat grain (100 g) was weighed into a 0.5 L glass bottle, and distilled water (50 mL) was added to the grain. The initial grain moisture was 13.8%. The bottles were closed with cellulose stoppers and autoclaved twice for 1 h at 121 °C. After cooling, the *Fusarium* isolates were inoculated onto the substrate with four mycelial discs per bottle. The control samples had the same treatment as described above but contained no fungal culture. The spring wheat grain was chosen as a substrate to simulate the natural habitat of the strains to obtain a better estimate of the toxin production in plants [50]. 

### 5.4. Storage Conditions

Spring wheat grain samples inoculated with *F. graminearum* isolates from different host plants were incubated in controlled climate chambers (Binder, Tuttlingen, Germany) for 5 weeks at 25 °C and 29 °C. 

After the incubation period, the grain cultures were air dried at 45 °C and ground to flour using an Ultra Centrifugal Mill ZM 200 (Haan, Germany) with a 0.8 mm sieve. Sample preparation for chromatographic analysis of mycotoxins was performed.

### 5.5. Sample Preparation for Mycotoxin Analyses 

The ground sample (2.50 ± 0.01 g) was added to a 50 mL PP tube and extracted with a mixture of deionized water (10 mL), acetonitrile (10 mL), and formic acid (20 μL) for 10 min on a mechanical shaker. After adding the QuEChERS salt mixture, samples were shaken for 10 min on a mechanical shaker. The samples were centrifuged for 10 min at 4000 rpm at room temperature. The extract was transferred to a 15 mL PP tube, placed in an ultra-low temperature freezer for 15 min at −80 °C, and, after removal, centrifuged immediately at 4000 rpm for 10 min at 10 °C.

A 3 mL portion of the extract was transferred to a 15 mL PP tube and evaporated to dryness at 50 °C under a stream of nitrogen. After evaporation, 0.1% formic acid in 1:1 acetonitrile-water solution (100 μL) was added, the solution was shaken on a Vortex mixer, and 0.1% aqueous formic acid solution (250 μL) was added. The extract was filtered through a 0.22 μm PVDF filter and centrifuged for 10 min at 3000 rpm at room temperature. Standard additives were added to the calibration and control samples in autosampler glass vials and evaporated to dryness under a gentle stream of nitrogen. Finally, 200 μL of the filtered extract was transferred to an autosampler glass vial with standard additives or without them.

### 5.6. Method of Analysis

The analysis was performed on an UltiMate 3000 (Thermo Fisher Scientific, Waltham, MA, USA) HPLC instrument coupled with a Thermo Scientific TSQ Quantiva MS/MS detector (Waltham, MA, USA). The separation was performed on a Phenomenex Luna C_18_ reversed-phase analytical column (150 × 2.0 mm, 3 µm). The autosampler was maintained at 4 °C, and the column temperature was 40 °C. The sample injection volume was 25 µL. Ion monitoring was conducted in both positive and negative ion modes, and the mass analysis was performed in the selected reaction monitoring (SRM) mode. The following instrumental settings were used: spray voltage 3.5 kV (positive ion mode), 2.5 kV (negative ion mode), vaporizer temperature 350 °C, ion transfer temperature 300 °C, sheath gas 55 arbitrary units (arb), auxiliary gas 25 arb, and sweep gas 5 arb. Data processing was performed with TraceFinder software (Thermo Fisher Scientific).

Phase A consisted of 0.1% formic acid, 0.5 mM ammonium acetate in water. Phase B consisted of 0.1% formic acid, 0.5 mM ammonium acetate in acetonitrile. 

### 5.7. Method Validation

Five-point calibration curves were constructed using blanks spiked with standard mycotoxin mixtures to evaluate the linearity. The least-squares regression method was used for slope construction and calculation of the determination coefficients (R^2^) of the calibration curves, which were evaluated to a fit of at least 0.99. For quality control purposes, the blank samples were spiked with standard mycotoxin solvents at the following concentration levels: 10, 50, and 100 µg kg^−1^ for DON, 3-ADON, 15-ADON, NIV, D3G, FUSX, and ZEA. The precision and accuracy were validated by analyzing five replicates at each of the three determined spiking levels. All method validation results are shown in Table 6. 

### 5.8. Statistical Analysis 

The reliability of the data was assessed using the statistical data processing program SAS Enterprise Guide 7.1. A one-way analysis of variance (ANOVA) statistical package was used to evaluate the data scatter and identify significant differences between data averages. Significant differences between the two samples were compared by using Duncan’s criterion. Additionally, the experimental data were analyzed using the statistical program R, version 3.6.0 (R Core Team, 2019, Vienna, Austria). A two-way analysis of variance (ANOVA) statistical package was used. The level of significance was set at *p* < 0.05, *p* < 0.01, and *p* < 0.001.

## Figures and Tables

**Table 1:** Mycotoxin occurrence in spring wheat grains depending on the temperature and trichothecene genotype.

		25 °C				29 °C			
Mycotoxin	Strain Genotype	Positive, %	Min, µg kg^−1^	Max, µg kg^−1^	Average	Positive, %	Min, µg kg^−1^	Max, µg kg^−1^	Average
DON	3-ADON	100	6804	101,100	37,013	100	286	15,473	3721
	15-ADON	100	8305	223,532	56,605	100	169	63,754	6577
NIV	3-ADON	43	<100	3015	866	14	<100	352	270
	15-ADON	19	<100	573	377	14	<100	162	129
D3G	3-ADON	28	<10	78	32	0	<10	<10	<10
	15-ADON	43	<10	187	51	5	<10	16	16
FUS-X	3-ADON	90	<10	160	60	28	<10	136	109
	15-ADON	100	11	152	39	33	<10	220	154
3-ADON	3-ADON	86	<50	33,344	13,954	86	<50	1150	407
	15-ADON	76	<50	11,899	3159	67	<50	1217	187
15-ADON	3-ADON	71	<50	2829	1133	57	<50	4739	1195
	15-ADON	95	<50	27,498	6815	67	<50	6658	1610
ZEA	3-ADON	100	10,995	52,763	25,805	100	219	43,150	9803
	15-ADON	100	15,290	46,686	29,771	100	2950	52,728	13,464

**Table 2:** ANOVA of the contribution of *F. graminearum* strains (Factor A) and incubation temperature (Factor B) to the production of mycotoxins on spring wheat grains.

		DON	3-ADON	15-ADON	NIV	D3G	FUS-X	ZEA
Factor A	*F*	4.632	8.012	7.295	1.643	0.989	1.050	8.575
	*p*	0.0124 *	0.000654 ***	0.00120 **	0.1996	0.3761	0.355	0.000409 ***
Factor B	*F*	37.681	26.306	9.408	3.864	5.939	0.000	42.816
	*p*	0.0000 ***	0.000000 ***	0.00291 **	0.0526	0.0169 *	0.994	0.00000 ***
Factor A x B	*F*	2.990	7.320	4.946	1.224	0.905	1.179	1.249
	*p*	0.0556	0.001174 **	0.00932 **	0.2992	0.4084	0.313	0.291984

*p < 0.01; **p < 0.001; ***p < 0.0001.

**Table 3:** The correlations between individual mycotoxins with trichothecene 15-ADON genotype (left) and with trichothecene 3-ADON genotype (right) in spring wheat incubated at 25 °C. Significant r values (*p* < 0.05) are in bold.

		DON	NIV	D3G	FUS-X	3-ADON	15-ADON	ZEA	
15-ADON genotype	DON		**0.696**	**0.791**	0.416	**0.603**	0.071	−0.266	3-ADON genotype
	NIV	0.099		**0.970**	**0.741**	0.171	−0.122	−0.306	
	D3G	0.018	**0.974**		**0.678**	0.229	−0.125	−0.456	
	FUS-X	0.078	**0.991**	**0.981**		0.481	−0.062	−0.028	
	3-ADON	0.230	0.087	0.022	0.093		0.210	0.157	
	15-ADON	**0.556**	0.002	−0.103	−0.020	**0.912**		**0.538**	
	ZEA	0.042	**−0.863**	**−0.787**	**−0.882**	−0.182	−0.038		

**Table 4:** The correlations between individual mycotoxins with trichothecene 15-ADON genotype (left) and with trichothecene 3-ADON genotype (right) in spring wheat incubated at 29 °C. Significant r values (*p* < 0.05) are in bold.

		DON	NIV	D3G	FUS-X	3-ADON	15-ADON	ZEA	
15-ADON genotype	DON		0.048	-	−0.261	**0.834**	−0.203	**0.582**	3-ADON genotype
	NIV	−0.157		-	0.473	−0.154	−0.119	−0.299	
	D3G	-	−0.202		-	-	-	-	
	FUS-X	−0.294	−0.317	−0.262		−0.089	0.381	−0.390	
	3-ADON	**0.984**	−0.095	**0.976**	−0.373		−0.147	**0.715**	
	15-ADON	−0.164	−0.294	−0.137	**0.967**	−0.237		−0.116	
	ZEA	**0.924**	−0.277	**0.934**	−0.403	**0.911**	−0.274		

**Table 5:** The study scheme.

Treatment No.	Host Plant	Strain Code	TRI Genotypes
1	Control		
2	*Fallopia convolvulus* (L.) Löve	144š	3-ADON
3	*Fallopia convolvulus* (L.) Löve	283š	15-ADON
4	*Viola arvensis* Murray	153l	15-ADON
5	*Viola arvensis* Murray	541s	3-ADON
6	*Triticum aestivum*	6K4V1	15-ADON
7	*Triticum aestivum*	B 45.4.1	3-ADON
8	*Brassica napus* L.	98p	3-ADON
9	*Brassica napus* L.	425l	15-ADON
10	*Euphorbia helioscopia* L.	762l	3-ADON
11	*Euphorbia helioscopia* L.	678v	15-ADON
12	*Tripleurospermum inodorum* (L.) Sch.	1120p	15-ADON
13	*Tripleurospermum inodorum* (L.) Sch.	1422p	3-ADON
14	*Poa annua* L.	787v	15-ADON
15	*Poa annua* L.	1350s	3-ADON

**Table 6:** Chromatography method validation parameters.

Validation Parameters										
Mycotoxin	LOD, µg kg^−1^	LOQ, µg kg^−1^	Linear Range, µg kg^−1^	R^2^	Accuracy (Deviation from the Theoretical Value, %)			Precision (RSD, %)		
					Level of Spiked Samples, µg kg^−1^					
					10	50	100	10	50	100
NIV	14	42	42–500	0.9983	x	−9	−18	x	9	3
D3G	4.5	13	13–500	0.9972	12	−3	−1	12	5	8
DON	4.0	12	12–500	0.9991	23	5	4	10	6	3
FUS-X	1.2	3.5	3.5–500	0.9988	6	7	0	3	6	8
15-ADON	14	42	42–500	0.9988	x	4	2	x	8	2
3-ADON	3.6	11	11–500	0.9991	−24	−11	−9	14	14	11
ZEA	2.4	7.1	7.1–500	0.9993	25	−10	−8	6	7	4

X—not found.

## Data Availability

Not applicable.
